# Effectiveness of Different Teaching Methods in Enhancing Dental Undergraduate Students’ Knowledge and Restorative Cement Manipulation Skills

**DOI:** 10.7759/cureus.67999

**Published:** 2024-08-28

**Authors:** Anju Varughese, Remya M, Deepthy S, Venkitachalam Ramanarayanan, Arya A Varghese, Vidya K G

**Affiliations:** 1 Department of Conservative Dentistry and Endodontics, Amrita School of Dentistry, Amrita Vishwa Vidyapeetham, Kochi, IND; 2 Department of Public Health Dentistry, Amrita School of Dentistry, Amrita Vishwa Vidyapeetham, Kochi, IND; 3 Department of Pediatric and Preventive Dentistry, Sri Ramaswamy Memorial (SRM) Kattankulathur Dental College, SRM Institute of Science and Technology, Kattankulathur, IND; 4 Department of Conservative Dentistry and Endodontics, Government Dental College, Thiruvananthapuram, Thiruvananthapuram, IND

**Keywords:** restorative materials, operative dentistry, knowledge, dental education, clinical competence

## Abstract

Background and objectives

Developing manual skills is important in dental education. Clinical skills can be taught via clinical demonstrations, and knowledge can be delivered through lectures. Performing dental restorations is an essential skill for dentists, which requires a comprehensive knowledge of the manipulation of the restorative materials. The present study sought to compare the effectiveness of flipped learning (FL), smart class (SC), and traditional teaching (TT) methods in two arenas of learning: acquisition of theoretical knowledge and practical skill in manipulating dental restorative cement.

Materials and methods

All first-year undergraduate dental students (n = 60) were divided into three study groups and exposed to three teaching methods, namely TT, SC, and FL of three different dental restorative cements. Each teaching method was followed by an evaluation of the “knowledge assessment score,” a live demonstration of the cement manipulation, and the participants’ “skill assessment score.” Descriptive statistics were expressed as mean and SD for continuous variables. A comparison of the knowledge assessment scores and skill assessment scores between the study groups was analyzed using a one-way ANOVA test. Intergroup comparison was done using Tukey’s post hoc test.

Results

The FL group had a significantly higher “knowledge assessment score” (p = 0.001), while there was no significant difference between the SC and TT groups (p = 1.0). Both FL and SC groups had significantly higher “skill assessment scores” (p = 0.001), with no significant difference between them (p = 0.798).

Conclusions

Of the three teaching methods assessed, FL proved to be more effective in the knowledge acquired and clinical competence demonstrated when compared to the other two techniques in dental education.

## Introduction

Technical abilities are taught and trained in dentistry through classroom lectures augmented by modern approaches like preclinical exercises, simulators, and Phantom models that simulate clinical scenarios [1]. Teaching modalities have drastically changed since the introduction of blackboards and chalk in the 1890s. Other didactic means, besides lecturing, were demanded to supplement teaching. Technologies such as overhead projectors, desktop computers, interactive whiteboards, and audiovisual aids were thus gradually introduced in the classroom.

In dental education, the development of dental operative skills is an important part of a student’s education [2]. This is achieved by various means like problem-based learning, case-based learning, computer-based e-learning, flipped classroom techniques, application of artificial intelligence, etc. These new approaches increase collaborative learning, critical thinking, creativity, problem-solving, communication abilities, and students’ self-confidence [3]. A systematic review by Khalifah and Celenza reported that educators combined passive and active strategies to provide education to enhance communication skills in clinical practice [4]. The traditional instruction course referred to as the all-in-one teaching method incorporated three tasks: theoretical lectures, practical demonstrations, and skill practice, which were conducted one after the other. However, the all-in-one method’s teaching effectiveness was not nearly sufficient, especially when a technique required multiple steps [2].

A critique of the traditional learning method is its teacher-centric approach. The involvement of the learner is a prerequisite for any professional course, which may be attained by implementing a learner-centered, effective teaching method. Thus, a shift from a traditional method to a learner-centered approach is critical [3].

Live demonstrations are effective at progressively explaining clinical procedures, allowing students to perform the skill on their own. It has been observed that for first-time learners, both live demonstrations and videotaped presentations of the same procedure were equally successful in conveying preclinical knowledge and clinical skills [1].

Gopinath and Nallaswamy [5] conducted a systematic review of several teaching strategies for dentistry students and evaluated their efficacy. The findings demonstrated that there is evidence to suggest that video-based teaching approaches improve dental students’ performance in preclinical procedures.

A cross-sectional study conducted among 324 third- and fourth-year medical and dental students reported that about one-third of the students chose standardized lecture slides with proper learning outcomes and well-explained by the lecturers as the best method. The study recommended a shift from the traditional teaching (TT) method to various approaches such as project-based learning (PBL), videos, quizzes, and conversations to meet educational goals; thus, teaching and learning become more interactive and effective [6]. PBL is an active and interactive learning approach that aims to guide students to build productive problem-solving skills, increase flexible knowledge, self-directed learning, successful cooperation skills, and intrinsic motivation [7,8].

Education has changed drastically in the contemporary period, with the advent of e-learning via digital platforms. According to research, online education has been demonstrated to effectively recall information in minimal time. Varvara et al. [9] conducted a questionnaire study to evaluate the perception of e-learning among dental undergraduate students. The new approaches and the attempts added by the lecturers to offer lectures of the highest possible quality were accepted by most students. However, a deficiency of practical training was identified as a critical issue in the design of their new curriculum.

In contrast to the didactic teaching model, a new model referred to as the “flipped classroom or inverted classroom” provides information on how to make the most of the time spent in class on critical thinking exercises to encourage students to maximize their opportunities and spend time outside of class performing collaborative exercises in the company of peers and instructors. Hu et al. proposed combining the flipped approach with PBL as a better alternative to the typical lecture-based classroom [10]. Flexibility, personalization (the opportunity to take up time on areas they do not grasp), and active learning are all advantages of a flipped classroom [11]. It aids in the development of improved learning skills, participation, and individual student achievement [3]. The flipped classroom approach has been accepted currently in health professions education and has been shown to yield a significant improvement in learner performance compared to TT methods [12].

When students are exposed to a wide range of teaching methods and have access to several methods for acquiring skills, the material is more likely to be understood, recalled, and reproduced [1]. Although many teaching methods have been used for educational application in dentistry, only limited research demonstrates the educational effectiveness of contemporary tools through experimental consideration. There is a paucity of literature on innovative teaching methods in the field of operative dentistry. Not much research has been done on the smart class (SC) and flipped learning (FL) in dental education in India.

In the Bachelor of Dental Surgery (BDS) curriculum, among other subjects, the students are introduced to dentistry through the subject of dental materials, where they are acquainted with various materials used in routine dental practice. This preclinical subject focuses on learning and training the students in the manipulation of materials like restorative cement and prosthodontic materials. At the end of the academic year, the students are assessed for their knowledge of dental materials through a theory evaluation and their manipulation skills via a practical examination.

Hence, this study intends to compare the effectiveness of FL, SC, and TT methods in two arenas of learning: acquisition of theoretical knowledge and practical skill in manipulating dental cement.

The preprint of this article can be accessed at https://doi.org/10.21203/rs.3.rs-4568409/v1 [13].

## Materials and methods

An educational intervention was designed for the undergraduate dental students. The study was approved by the Institutional Review Board of the Amrita Institute of Medical Sciences, Kochi, India (approval number IRB-AIMS-2020-104). The study was conducted from April 2022 to June 2022 at Amrita School of Dentistry, Kochi, India.

The cement manipulation is a part of the preclinical curriculum, which students are expected to perform in the first academic year. As this was an exploratory study, all the first-year BDS students were included in the study (n = 60). Informed consent was obtained from all the study participants.

Three different theory topics of dental materials on restorative cement were selected for the lecture: zinc oxide eugenol cement, zinc phosphate cement, and zinc polycarboxylate cement. The participants had no prior idea about any of these types of dental cement and its material sciences. The study evaluated the different teaching methods: TT, SC, and FL.

TT, lecturing with the aid of a blackboard and chalk, is the most widely used method at all levels of education. SC, the prime mode of teaching in educational institutions, is a lecture tutorial delivered with audiovisual aids. In the FL technique, the study material related to the topic was given to the concerned group of students. They were given an hour to read and discuss among themselves before the lecture with audiovisual aids. The videos used for the demonstration were recorded by the instructors. The handouts and pictures used in the study were taken from standard textbooks followed by the students.

All the 60 study participants were randomly divided into three groups (n = 20) using the lottery method (fish bowl technique): group A (G-A), group B (G-B), and group C (G-C) and assigned to the intervention as given in Table 1.

**Table 1:** Order of teaching methods for each cement FL, flipped learning; SC, smart class; TT, traditional teaching; ZOE, zinc oxide eugenol; ZPC, zinc polycarboxylate; ZPO, zinc phosphate

Topic		TT	SC	FL
Day 1	ZOE	G-A	G-B	G-C
Day 2	ZPO	G-B	G-C	G-A
Day 3	ZPC	G-C	G-A	G-B

The theory classes were taken on three consecutive days. Each theory class, of 30 minutes, was followed by a live demonstration of the concerned cement. The order of teaching methodology for each cement is given in Table 1. The teaching methodology was planned in each group for each cement in such a manner that each student would be exposed to three different teaching methods. To prevent any form of interaction between the groups, the participants of each group were made to remain in three different classrooms while the students were unaware of the topic for the day. Only the students in the FL group were provided with the handout.

The theory classes of all three types of cement of three different teaching methods (a total of nine sessions) were taken by a single instructor to avoid bias. Each teaching method is followed by evaluating the “knowledge assessment score” using a multiple-choice objective question response test of similar difficulty level developed by a single investigator to avoid bias. It had 10 questions carrying one mark each that needed to be answered at the end of every class by the students to determine the “knowledge assessment score” (Appendix A).

Once all three groups were done with the theory classes for a particular cement, a live demonstration of cement dispensing, proportioning, and manipulation was given by a second instructor for all the groups to avoid bias. After all the lecture classes and the live demonstrations, each student had to perform the cement manipulation of all three types of cement. The manipulation skills were assessed based on certain criteria stated in the standard textbook [14], and a scoring system was used to evaluate the study participants for all three types of cement. The criteria include powder: liquid ratio, proportioning, manipulation technique used, mixing time, and consistency of the mix, with a score of 1 (correct) or 0 (wrong) per criteria with a maximum score of 5. It was monitored, and the “skill assessment score” was recorded by a third instructor, who was blinded to the intervention, to avoid detection bias in outcome assessment. The first, second, and third instructors were calibrated, and the kappa statistical value was noted to be 90.6, 92.3, and 86.7, respectively.

The observed data were coded, tabulated, and analyzed using IBM SPSS Statistics for Windows, Version 20.0 (Released 2011; IBM Corp., Armonk, NY, USA). Descriptive statistics were expressed as mean and SD for continuous variables. The normality of the data was assessed using the Shapiro-Wilk test, which was found to be parametric. Therefore, the comparison of the knowledge assessment scores and skill assessment scores between the study groups was done using a one-way ANOVA test. Intergroup comparison was done using Tukey’s post hoc test.

## Results

A total of 60 students were enrolled in the study and designated into three groups of 20 each. One student from each of the three groups had to be excluded from the final analysis as they missed one of the theory classes. Thus, the final analysis was based on 57 participants (19 in each group).

There was a statistically significant difference in the knowledge assessment score (out of 10) between the three study groups, with the FL group exhibiting the highest scores (8.11 ± 1.08) and TT and SC showing near similar scores (Table 2). The FL method was noted to be effective in enhancing the understanding and the learning ability of the students.

**Table 2:** Comparison of knowledge assessment scores between study groups FL, flipped learning; MCQ, multiple choice objective question; SC, smart class; TT, traditional teaching

Outcome	Teaching method	N	Min	Max	Mean ± SD	p-value
MCQ / 10	TT	57	5	9	6.30 ± 1.23	<0.001*
	FL	57	6	10	8.11 ± 1.08	
	SC	57	5	8	6.35 ± 0.95	
	Total	171	5	10	6.92 ± 1.37	

On post hoc analysis, it was observed that statistically significant differences were observed between FL-TT groups and FL-SC groups (p < 0.001). No significant differences were found between SC-TT groups (p = 1.000) (Table 3).

**Table 3:** Post hoc analysis of knowledge assessment scores between study groups FL, flipped learning; SC, smart class; TT, traditional teaching

Dependent variable	(I) Teaching method	(J) Teaching method	Mean difference (I-J)	p-value
MCQ / 10	TT	FL	-1.807	<0.001*
		SC	-0.053	1
	FL	TT	1.807	<0.001*
		SC	1.754	<0.001*
	SC	TT	0.053	1
		FL	-1.754	<0.001*

A statistically significant difference also existed for the skill assessment scores (out of 5), with the FL group performing the best (3.86 ± 0.66) (Table 4). The participants who received the FL method had the highest score and the least than the ones who received the TT method. The FL method was noted to be effective in enhancing the restorative cement manipulating skills of the students.

**Table 4:** Comparison of skill assessment scores between study groups FL, flipped learning; SC, smart class; TT, traditional teaching

Outcome	Teaching method	N	Min	Max	Mean ± SD	p-value
Restorative cement manipulation / 5	TT	57	2	3	2.56 ± 0.50	<0.001*
	FL	57	3	5	3.86 ± 0.66	
	SC	57	3	5	3.74 ± 0.58	
	Total	171	2	5	3.39 ± 0.82	

On multiple comparisons, the TT group (2.56 ± 0.50) was found to be significantly lower than the FL (3.86 ± 0.66) and SC groups (3.74 ± 0.58) (p < 0.001). No significant differences were found between the FL and SC groups (p = 0.798) (Table 5). Both FL and SC methods were effective in enhancing the restorative cement manipulation skill.

**Table 5:** Post hoc analysis of skill assessment scores between study groups FL, flipped learning; SC, smart class; TT, traditional teaching

Dependent variable	(I) Teaching method	(J) Teaching method	Mean difference (I-J)	p-value
Restorative cement manipulation / 5	TT	FL	-1.298	<0.001*
		SC	-1.175	<0.001*
	FL	TT	1.298	<0.001*
		SC	0.123	0.798
	SC	TT	1.175	<0.001*
		FL	-0.123	0.798

## Discussion

With increasing awareness of the limitations of routine memorization learning, the idea of fostering self-directed learning in students has emerged. Of all the methods that are applied in medical education, the flipped classroom has proved to enhance students’ ability to debate various topics, their cooperative learning ability, and most of all, their self-directed learning ability [15]. The use of flipped class learning among the students showed a significant improvement in the assimilation of knowledge about the respective subject. The students are exposed twice to the same content with a period to discuss with their peers, boosting their interest and understanding of the subject.

“Flipping the classroom” means that students gain first exposure to new material outside of class, usually via reading or watching lecture videos, and then use class time to assimilate that knowledge through problem-solving, discussion, or debates in the presence of an instructor or facilitator [16]. Mazur popularized the paradigm, saying that it focuses on student and interactive learning and increases learning gains almost threefold [17]. As the FL technique is advocated, in the classroom environment, which is a large learning group, the individual has the opportunity to experience learning with their classmates and also internalize these experiences individually. From the perspective of these assumptions, the flipped classroom is a teaching model that is suitable for an active learning approach [18].

The present study sought to evaluate the effectiveness of FL in comparison to the TT methods and SC in two arenas of learning; acquisition of theoretical knowledge and practical skill. The results showed maximum scores in the knowledge test among students who followed FL, which was statistically higher than the other methods. The SC and TT, however, showed no significant differences. The results were consistent with the findings by Behmanesh et al. [19]. who demonstrated significant improvement with FL over TT in improving midwifery students’ knowledge and practice. When FL was used in the pediatric dentistry course with students from a predoctoral program, Gallardo et al. demonstrated an improvement in course grades; however, this improvement was not statistically significant [20]. A similar study by Bohaty et al. [21], although showing improved scores with a flipped classroom model, did not mention if the findings were significant or not. Other studies by Xiao et al. [22] based on a quiz post-lecture on the module taught on physiology, Lee and Kim [23] based on a knowledge quiz on periodontal diagnosis and treatment planning, and Kavadella et al. [24] analyzed the results of the questionnaires and the tests pre- and post-lecture on oral radiology and showed improved student learning outcomes in the flipped classroom design. This outcome may be attributed to several factors. Firstly, the students are exposed to the teaching material before the class, allowing them to prepare well before the class. Secondly, it provides a platform to discuss with their peers, which boosts their interactive and critical thinking skills. This was studied and concluded by Shi et al. [25]: students can develop high-level cognitive thinking abilities through FL.

Finally, the active participation of students elevates the interest in the subject to a deeper level, unlike seen in the one-sided classic didactic lectures. Vanka et al. [26] reported a majority of the students hold a positive opinion of the flipped classroom model, reflecting an increased level of student engagement. Chowdhury et al. [27] reported positive feedback from undergraduate students, which showed they valued case-based interactive discussions but could not demonstrate an improvement in assessment scores. Riddell et al. [28] found essentially equivalent scores in a crossover study comparing a single flipped classroom module with a standard lecture in emergency medicine programs. The systematic review by Karabulut-Ilgu et al. [29] and the study by Elledge et al. [30] indicated that students in the flipped classroom had learned as much as their counterparts taught by traditional lectures with no significant difference.

Concerning the improvement in practical skills, a better outcome was evident with flipped classrooms and SC teaching over the TT method. However, there was no difference between the SC and flipped classroom techniques. This points to the advantage of using audiovisual aids. This concept was also proposed by Gallardo et al. [20], who stated that preclinical classes, when supplemented with pre-access videos that represent the actual procedure, can improve student learning. Crothers et al. [31] conducted a study to evaluate the flipped classroom for preclinical dental skills teaching and demonstrated many advantages of this model for preclinical skills teaching, as reflected in very positive feedback from students. Thus, FL proved to be more effective when compared to other techniques, both knowledge acquired and practical skills demonstrated.

The students enrolled in the present study had all passed a professional examination for admission, which assumes an above-average IQ for all students. However, a prior evaluation test may be conducted to evaluate the IQ level of each student, which may influence the grasping capacity of each student. This was further emphasized by Kim [15], who suggested that dental instructors should carefully develop and apply more learner-based facilitation and relevant assessment by considering student learning styles and personality types before implementing a flipped classroom approach. Previous studies have indicated the different impacts pre-classroom learning materials may have on the effectiveness of flipped classroom teaching [5,32,33]. Approximately 60 minutes of preparatory time has been recommended but not rigorously evaluated, particularly in medical student education [33].

The present study utilized reading material pertaining to the curriculum. However, other means may also have been used, and possible differences in effect may be compared. As this was an exploratory study, only one institution was included. We recommend further studies in different contexts with larger sample sizes. Another limitation of the present study was the assessment of knowledge and practical skills, which was conducted shortly after the delivery of content, and a long-term evaluation was not performed. Future studies may evaluate the ability of students to foster knowledge and skills over long periods to assess the effectiveness of the different teaching methods more beneficially.

This study demonstrates that FL is superior to TT and SC in enhancing knowledge retention and skill acquisition in dental students. These results suggest that incorporating FL into dental curricula could significantly enhance student outcomes, particularly in practical skill development.

## Conclusions

The comparison of the mean knowledge score of the three teaching methods showed that FL and SC are more effective when compared to traditional blackboard and chalk teaching methods. Skill enhancement was comparatively better when the FL technique was used.

The results of the present study imply the need for a blended teaching methodology that helps to improve their knowledge and operative skills for effective dental practice. Of the three teaching methods assessed, FL proved to be more effective when compared to other techniques, with both knowledge acquired and practical skills demonstrated. Classroom learning can be improved to be more student-centered with effective teaching methods. Given the significant improvement in skills with FL, dental schools should consider integrating novel methods into the curriculum, specifically for hands-on skills training.

## Figures and Tables

**Figure 1:**
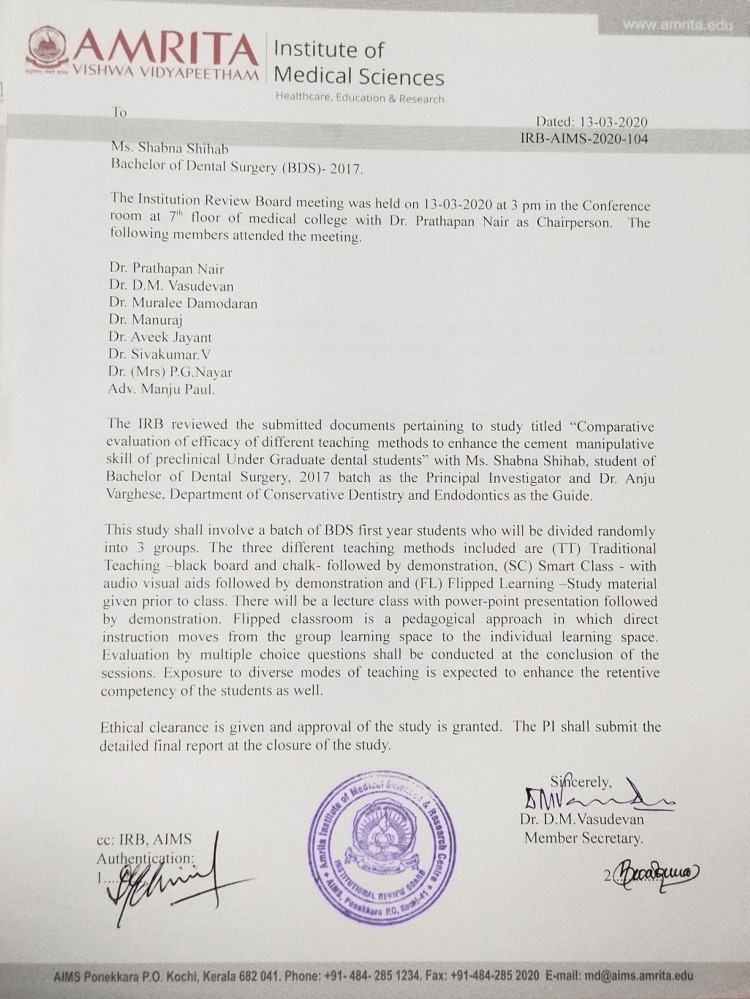
IRB approval

## Appendices

Appendix A

Theory 1: ZOE

MCQ

1. The addition of which of the following can accelerate the setting time of zinc oxide cement:

a) Zinc acetate     b) Barium sulpate  c) Zinc phosphate         d) barium chloride

2. Eugenol may be replaced in the zinc oxide eugenol cement by:

a)   Acetic acid     b) Alginic acid    c) Phosphoric acid d) ortho-ethoxy acid

3. Resins are incorporated in zinc oxide eugenol cement to:

a) Decrease strength     b) decrease film thickness          c) increase strength       d) none of the above

4. which of the following is most important in strength of ZOE:

a) powder liquid ratio   b) temperature of a mixing slab      c) speed of mixing

d)Addition of a few drops of water

5. The effect of zinc oxide eugenol cement on the pulp?

a) is irritating      b) Encourages pulpal fibrosis        c) is sedating        d) Has no effect

6. The percentage of zinc in ZOE cement is:

a) 60 %          b)70%            c) 80 %             d)90 %

7. most preferable cement for temporary restoration

a) GIC            b) ZOE            c) Zinc polycarboxylate           d) calcium hydroxide

8. Zinc oxide eugenol is not used for permanent cementation because of.

 a) high solubility        b) poor marginal seal       c) poor pulp protection         d) less strength

9. Zinc oxide powder reacts with eugenol to form zinc eugenolate.it is an example of:

a) polymerization reaction b) decomposition reaction c) chelation reaction d) none of the above

10. About zinc oxide eugenol all are true except -

a) Zno is converted to Zinc hydroxide        b) reaction is autocatalytic       c) water is the byproduct of

 the reaction       d) Dehydrated ZnO  reacts  with  dehydrated  eugenol

Theory 2: ZPO

1 Minimum thickness of type 1 Zinc phosphate cement should be:

a) 15 microns     b) 25 microns      c) 50 microns     d) 100 microns

2.  Increased amount of powder in zinc phosphate cement mixture will cause:

a) Decreased strength b) Decreased film thickness c) Decreased solubility d) Increased setting time

3. pH of fully set zinc phosphate:

a) 3-4        b) 4-5        c) 6-7         d) 7-8

4. The setting time of zinc phosphate may be retarded by:

a) Increase in the ratio of powder to liquid       b) Diluting the liquid with water

c) Increase the addition of powder to liquid     d) Decrease the addition of powder to liquid

5) The chief advantage of zinc phosphate cement is its:

a) good compressive strength     b) film thickness       c) lack of irritation         d) low solubility 

6) The major component of zinc phosphate cement is:

a) zinc acetate        b) phosphoric acid           c) zinc oxide          d) magnesium oxide

7. The principal application of zinc phosphate cement is:

  a) In final cementation                            b) As temporary cementation       

  c) As a temporary filling material            d) As a permanent restoration

8) Which cement base has the highest modulus of elasticity?

a) zinc polycarboxylate      b) polymer-reinforced ZOE cement      c) zinc phosphate         d) GIC

9. Frozen slab technique of mixing zinc phosphate is used for :

a) cementing    crowns       b) temporary dressing       c) base  d) cementing orthodontic bands

10. The Advantage of zinc phosphate over GIC is :

a) High compressive strength            b) Tensile strength  

 c) High modulus of elasticity             d) Diametral strength

Theory 3: ZPC

MCQ

1. Powder liquid ratio of zinc polycarboxylate cement:

a)  1:1         b) 2:1            c)  1.5:1            d)  2.5:1

2. Which of the following shows a chemical bond with enamel and dentin:

a) Composite    b) Zinc phosphate    c) Glass ionomer cement         d) Calcium hydroxide

3. Which of the following do polycarboxylate and GIC have in common?

a) Polysiloxane      b) Phosphoric acid    c) Polyacrylic acid     d) Ion leachable glass

4. pH of polycarboxylate liquid  prior to manipulation of the cement  :

a)  2. 5      b) 2. 7          c)    5       d)    1.7

5. Zinc polycarboxylate is less irritant to the pulp due to its:

a)  pH          b) composition        c) particle size           4) setting reaction

6. polycarboxylate cement is used for :

a) Temporary restoration    b) Cementation     c) Permanent restoration        d) Die material

7 . Minimum strength required for a base under restoration is :

a) less than 0.5 mpa      b) more than 1.2 mpa       c) 0.5 to 1.2 mpa            d) none of the above

8. Secondary caries is least likely seen with:

a) Silicate cement     b) Zinc phosphate        c) Zinc phosphate         d) GIC

9. The mechanism of adhesion of GIC restoration with tooth surface is by means of  ?

a) c =c double bond       b) chelate with metal ions       c) carboxyl group        d) polymer chains

10. The greatest amount of fluoride recharge is seen with:

a) Composite     b) Resin-modified glass ionomer cement     c) Resin composite     d) Polycarboxylate

IRB approval letter
